# When illustrations mislead: cognitive load and the simple view of reading in scientific text learning

**DOI:** 10.3389/fpsyg.2026.1952263

**Published:** 2026-10-01

**Authors:** Qinhan Zhang, Wenzhe Zhang, Rongfang Yang, Mengran Yuan, Ying Wang

**Affiliations:** 1 School of Psychology, Zhejiang Normal University, Jinhua, Zhejiang, China; 2 College of Art, Zhejiang Normal University, Jinhua, Zhejiang, China

**Keywords:** cognitive load, misleading illustrations, multimedia learning, reading comprehension, scientific text learning, simple view of reading

## Abstract

**Introduction:**

Scientific text learning often uses instructional illustrations to support learners’ understanding of abstract concepts, processes, and causal mechanisms. However, illustrations may mislead learning when they resemble conceptual illustrations but contain incorrect mechanistic information. Drawing on multimedia learning theory, cognitive load theory, and the simple view of reading, this study examined how misleading illustrations affect learning from short scientific expository texts involving causal mechanisms and whether cognitive load and language-related abilities explain these effects.

**Methods:**

Using a randomized three-condition between-subjects experimental design, 179 learners were assigned to conceptual-illustration, misleading-illustration, or plain-text conditions.

**Results:**

Compared with plain text and conceptual illustrations, misleading illustrations significantly reduced inference and overall learning performance. Mediation analysis showed that this negative effect was partly explained by increased cognitive load. Moderation analysis further indicated that reading comprehension consistently buffered the detrimental effects of misleading illustrations, whereas decoding ability did not show a comparable protective role. Item-level mixed-effects models supported the robustness of these findings.

**Discussion:**

These findings suggest that, within the short scientific expository texts examined here, mechanistic consistency and reading comprehension may represent important conditions shaping effective text–picture integration.

## 1 Introduction

Scientific texts frequently combine verbal explanations with visual representations to communicate abstract concepts, processes, and causal mechanisms that are difficult to observe directly. Such illustrations are generally intended to support comprehension by helping learners organize and integrate information across representational formats. However, visual information is not necessarily beneficial for learning. When an illustration appears explanatory but conveys information that is inconsistent with the mechanism described in the text, it may instead interfere with learners’ construction of coherent scientific understanding. Given the widespread use of illustrations in textbooks and digital learning materials, understanding the consequences of inaccurate visual representations is increasingly relevant to instructional design. Examining such cases may help clarify the conditions under which visual representations facilitate, rather than hinder, scientific understanding.

### 1.1 Instructional illustrations in scientific text learning

Scientific text learning is an important pathway through which learners understand natural phenomena, acquire scientific concepts, and develop explanatory understanding. Compared with narrative texts, scientific texts typically contain more abstract concepts, technical terms, process relations, and causal mechanisms. Learners are therefore required not only to retrieve explicitly stated information, but also to integrate distributed concepts and relations into coherent mental representations, thereby constructing explanatory understanding of scientific phenomena (Kintsch, 1998; McNamara, 2004; Ozuru et al., 2009). This is particularly important in natural science topics, where many key processes and internal mechanisms cannot be directly observed. Learners often need to rely on textual descriptions and external representations to understand structural relations, process sequences, and causal chains (Cook, 2006; Graesser et al., 2002).

Instructional illustrations are a central component of scientific text materials. Science textbooks and digital learning resources widely use diagrams, process illustrations, structural figures, photographs, and model-based representations to help learners understand scientific content that is difficult to convey through text alone. Early research on text illustrations suggested that illustrations may serve different functions, including decorative, representational, organizational, interpretive, and transformational functions (Carney and Levin, 2002; Levin et al., 1987). Among these, illustrations that support deep understanding are typically those that represent structural relations, process relations, or causal relations (Levie and Lentz, 1982). From the perspective of multiple external representations, visual representations can also support learning by complementing textual information, constraining interpretation, and promoting deeper knowledge construction (Ainsworth, 2006; Cook, 2006).

With the increasing use of digital and multimodal learning materials, learners are expected not only to comprehend written information, but also to understand how images present, organize, and imply meaning. Research on visual literacy and multimodal reading has emphasized the close relationship between image comprehension and text reading, suggesting that learners need to establish meaningful connections among text, images, and contextual information to construct stable understanding from multimodal materials (Farrar et al., 2024). Thus, in scientific text learning, illustrations are not merely supplementary visual elements; rather, they function as important external representations that support learners’ construction of conceptual relations and scientific explanations.

However, learners do not automatically benefit from illustrations. The effectiveness of text–picture learning depends on whether learners can establish appropriate mappings between textual and pictorial representations and integrate them into a coherent mental model (Schnotz and Bannert, 2003). In an eye-tracking study of science textbook learning, Hannus and Hyönä (1999) found that students did not always make sufficient use of illustrations, whereas higher-ability learners attended more strategically to relevant textual and pictorial information. Similarly, Hegarty and Just’s (1993) study of mechanical system comprehension showed that the benefits of combining text and diagrams depend on whether both representations support the same comprehension goal. More recent research has provided further evidence for the importance of active text–picture integration. Latini et al. (2020) found that integrative processing, reflected in transitions between complementary textual and pictorial information, was positively associated with comprehension of illustrated texts. More directly, Frick and Schüler (2023) showed that learners engage in activation, integration, and validation processes when processing illustrated texts, with inconsistent information imposing additional processing demands.

Therefore, whether illustrations facilitate scientific text learning depends not simply on the presence of visual materials, but on whether the illustrations accurately represent the structures, processes, and causal relations required by the learning goal.

### 1.2 Misleading illustrations: learning risks arising from erroneous visual explanations

The instructional value of illustrations depends on whether they effectively support learners in constructing accurate mental representations. Previous research has shown that visual materials can either facilitate or interfere with comprehension (Ainsworth, 2006; Carney and Levin, 2002; Cook, 2006; Schnotz and Bannert, 2003). Studies on decorative pictures and seductive details have found that vivid visual information that is weakly related to learning goals may attract learners’ attention but reduce their processing of core content (Harp and Mayer, 1998; Rey, 2012; Schneider et al., 2016; Sung and Mayer, 2012). However, the misleading illustrations examined in the present study differ from merely decorative or weakly relevant pictures. They are not simply irrelevant to the learning goal; rather, they resemble conceptual illustrations in form while conveying misleading explanatory information.

Research on misleading data visualizations provides an important basis for understanding this type of visual risk, in which information appears plausible but is nonetheless erroneous. Recent studies have examined design flaws in visualizations and their effects on comprehension from different perspectives. Lan and Liu (2025), for example, analyzed visualization design flaws from the public’s perspective and showed that such flaws may involve incorrect information, unclear labels, distorted proportions, inappropriate visual encoding, or ambiguous relational representations. Lo and Qu (2025) similarly argued that poorly structured visualization designs may affect readers’ judgments of graphical information, highlighting the need for clearer explanations to help readers identify and correct design problems. In addition, Svalina et al. (2024) found that three-dimensional depth cues in graphs influenced viewers’ gaze patterns and comprehension of bar charts. Together, these studies suggest that specific design features in visual materials can alter how learners interpret informational relations. Rho et al. (2026) further compared multiple types of misleading data visualizations and found that different misleading visual features varied in their effects on interpretation accuracy. Importantly, some misleading features continued to impair comprehension even among learners with higher data literacy. At the same time, research on data and visual literacy education has increasingly emphasized that learners need not only to read visual information, but also to evaluate whether visual representations are accurate, clear, and trustworthy (Dorsey et al., 2025). These recent findings suggest that the key risk of misleading visual materials is not merely whether they are relevant, but whether they present erroneous informational relations in a visually credible form.

Erroneous conceptual illustrations may have a similar misleading effect in scientific text learning. Recent research on misleading graphs and visualization design flaws indicates that errors in visual materials do not always appear as obvious falsehoods; they may also influence readers’ judgments of informational relations through labels, visual encoding, or graphical features (Driessen et al., 2022; Lan and Liu, 2025; Lo et al., 2022; Rho et al., 2026). For example, specific visual features such as three-dimensional depth cues can shape how readers attend to and understand graphs (Svalina et al., 2024). By extension, in scientific text learning, if a conceptual illustration resembles an explanatory illustration in form but conveys information that is inconsistent with the mechanism described in the text, learners may integrate these erroneous visual cues into their understanding of the text. In this case, misleading illustrations do not interfere with learning simply because they are unrelated to the text. Rather, they are problematic because they provide incorrect conceptual cues in the form of explanatory illustrations. Accordingly, the present study defines misleading illustrations as instructional illustrations that resemble conceptual illustrations in explanatory form but contain incorrect information about key conceptual relations, process relations, or causal mechanisms.

However, prior research leaves an important tension unresolved regarding whether learners can resist misleading visual information. On the one hand, data literacy and related visual-evaluation abilities may help learners critically evaluate visual information and reduce their susceptibility to misleading visualizations (Dorsey et al., 2025; Rho et al., 2026). On the other hand, recent findings suggest that some misleading visual features continue to exert detrimental effects even among learners with relatively high literacy levels (Driessen et al., 2022; Rho et al., 2026). This suggests that the effects of misleading visual information cannot be explained simply by whether learners are capable of “reading” a visual display. Instead, it is necessary to distinguish which cognitive abilities play a protective role in specific learning contexts.

This issue is particularly important for scientific text learning, where resisting misleading visual information may require learners to construct a stable situation model from the text and monitor its consistency with the accompanying illustration. Therefore, the present study draws on the simple view of reading to distinguish decoding ability from reading comprehension and to examine whether these two language-related abilities differentially shape learners’ resistance to misleading illustrations that misrepresent conceptual structures, process sequences, or causal mechanisms rather than primarily distorting quantitative relations.

### 1.3 The moderating role of language-related abilities from the perspective of the simple view of reading

The simple view of reading provides a useful framework for addressing the tension discussed above. According to this view, reading comprehension is jointly determined by decoding ability and linguistic comprehension, although these two components play different roles in reading processes (Gough and Tunmer, 1986; Hoover and Gough, 1990). Decoding ability primarily supports learners in recognizing written words and entering text-based processing, whereas reading comprehension involves higher-level processes such as semantic integration, discourse coherence, and the understanding of implicit relations. Recent reading research has also emphasized that reading comprehension is not merely the outcome of word recognition, but involves multiple levels of processing, including vocabulary, background knowledge, discourse integration, and inference making (Capin et al., 2025; Kintsch, 1998; Perfetti and Stafura, 2014).

In text–picture learning contexts, both decoding ability and reading comprehension may influence how learners process instructional materials. Haavisto et al. (2025) extended the simple view of reading to learning from illustrated texts and videos, showing that decoding ability and reading comprehension may affect learning outcomes and cognitive load in different ways. In the present study, all learning conditions involved written texts; therefore, decoding ability was expected to serve as a basic prerequisite for understanding the materials. However, the central challenge posed by misleading illustrations may not lie only in whether learners can recognize written words. Rather, learners also need to construct an accurate situation model from the text and evaluate whether the mechanistic information presented in the illustration is reliable.

Thus, both decoding ability and reading comprehension may be related to learners’ resistance to misleading illustrations, but they may operate through different pathways. Learners with stronger decoding ability may enter text processing more fluently and reduce the cognitive resources required for basic reading processes. In contrast, learners with stronger reading comprehension may be better able to identify the main ideas of the text, integrate core causal relations, and maintain a stable text-based representation when visual information is erroneous. In other words, resisting misleading illustrations may require not only efficient word recognition, but also higher-level discourse integration and monitoring of text–picture consistency. Accordingly, the present study distinguished decoding ability from reading comprehension and examined whether these two language-related abilities differentially moderated the effects of misleading illustrations on scientific text learning.

### 1.4 Cognitive load as a process mechanism linking misleading illustrations to learning

Misleading illustrations may affect scientific text learning by increasing cognitive load. Cognitive load theory assumes that learners have limited working memory resources, and that the complexity, presentation format, and irrelevant information in learning materials can influence the cognitive resources available for understanding and integrating core content (Paas et al., 2003; Sweller, 1988; Sweller et al., 2011). In multimedia learning, well-designed visual materials may reduce learners’ search, inference, and integration costs. In contrast, inappropriate visual presentations may increase extraneous cognitive load by requiring learners to allocate limited cognitive resources to unnecessary or erroneous information (Mayer, 2021; Mayer and Moreno, 2003).

Recent research has begun to examine the cognitive consequences of erroneous or inconsistent visual information, including its effects on cognitive load and additional processing demands. Dechamps and Skulmowski (2025), for example, examined erroneous instructional visualizations and subjective extraneous cognitive load. Related studies of text–picture inconsistency have further shown that conflicting textual and pictorial information can alter learners’ processing behavior and require additional integration and validation processes (Frick and Schüler, 2023; Schüler, 2017). Together, these studies suggest that when visual information conflicts with or misrepresents the information conveyed by the text, learners may need to devote additional cognitive resources to processing and reconciling the two representations. However, direct empirical evidence specifically linking misleading instructional illustrations to cognitive load remains limited.

This additional processing requirement may be particularly relevant to misleading illustrations. Learners need not only to understand the text itself, but also to process the visual cues provided by the illustration. When an illustration accurately represents the key structures and causal relations in the text, it may help learners organize information and reduce interpretive costs. However, when an illustration contains incorrect mechanistic information, learners may either treat the erroneous relations as valid explanatory cues or engage in additional processing to evaluate and reconcile the relation between textual and visual information. Such additional processing may increase learners’ subjective cognitive burden and consume cognitive resources that would otherwise be available for understanding the core scientific content.

Thus, the negative effects of misleading illustrations may arise not simply because they fail to provide useful information, but because they increase the processing costs involved in understanding and integrating the learning materials. In this sense, cognitive load may serve as an important process mechanism linking misleading illustrations to reduced learning performance.

### 1.5 The current study

Although previous studies have examined misleading visualizations and text–picture inconsistency, relatively little is known about how mechanistically misleading illustrations affect learning from scientific texts (Driessen et al., 2022; Frick and Schüler, 2023; Rho et al., 2026). Moreover, direct evidence regarding the role of cognitive load in this process remains limited, and it is still unclear whether decoding ability and reading comprehension differentially protect learners from misleading visual information (Dechamps and Skulmowski, 2025; Haavisto et al., 2025). These gaps highlight the need to examine both the cognitive mechanism and individual differences underlying the effects of misleading illustrations.

Building on these issues, the present study examined the effects of misleading illustrations on learning from two short scientific expository texts and further investigated the roles of cognitive load and language-related abilities in this process. Specifically, we used scientific expository texts as learning materials and created three instructional conditions: conceptual illustration, misleading illustration, and plain text. In the conceptual-illustration condition, the illustrations accurately represented the key structures and causal mechanisms described in the text. In the misleading-illustration condition, the illustrations were similar in form to the conceptual illustrations but contained incorrect mechanistic information. In the plain-text condition, no illustrations were provided. This design allowed us to distinguish the effects of accurate explanatory visual support from those of erroneous explanatory visual cues on short scientific text learning.

The current study addressed four research questions. First, do different types of instructional materials affect learners’ retention, inference, and overall learning performance? Second, do misleading illustrations impair learning performance by increasing cognitive load? Third, from the perspective of the simple view of reading, do decoding ability and reading comprehension differentially moderate the effects of instructional materials on learning performance? Fourth, are these effects robust at the item level?

## 2 Method

The study employed a randomized three-condition between-subjects experimental design. Participants were randomly assigned to the conceptual-illustration, misleading-illustration, or plain-text condition. Each participant studied both scientific text topics under the same assigned instructional condition, and the presentation order of the two texts was counterbalanced to control for potential order effects.

### 2.1 Participants

The final sample consisted of 179 valid participants. Participants were randomly assigned to one of three instructional conditions: conceptual illustration (*n* = 59), plain text (*n* = 60), or misleading illustration (*n* = 60). Participants ranged in age from 18 to 26 years (M = 21.17, SD = 1.54). Among them, 67 were male and 112 were female.

All participants completed the experiment through an online experimental platform. Before the experiment, participants read the study instructions and provided informed consent. They received monetary compensation after completing all experimental tasks and passing the data-quality screening. Data were excluded if participants failed to complete key measures, had missing response records, or showed response patterns that clearly did not meet the experimental requirements.

Ethical compliance was maintained in accordance with the Declaration of Helsinki and relevant institutional standards. Participants were assured of anonymity and were informed that they could withdraw from the study at any stage without penalty. Data were collected in March 2026 through a secure online experimental platform. The study was approved by the Ethics Committee of University (ZSRT2026205).

### 2.2 Materials

#### 2.2.1 Reading texts and illustration manipulation

The experimental materials consisted of two expository science texts on the topics of volcanoes and glaciers. The texts were adapted from Geography and Life (Getis et al., 2017). Each text was approximately 600–800 Chinese characters in length. Both texts were written in an expository style and focused on causal mechanisms, conceptual relations, and basic scientific principles underlying natural phenomena. To ensure comparability across topics, the two texts were structured around core concepts, formation mechanisms, typical features, and scientific explanations.

Following the material construction approach of Jaeger and Wiley (2014), three versions of the learning material were created for each text: a conceptual-illustration version, a misleading-illustration version, and a plain-text version. The conceptual-illustration version included diagrams that were closely integrated with the core content of the text, such as a diagram of volcanic eruption mechanisms and a diagram of glacier movement processes. These illustrations were designed to represent the key causal and structural relations described in the text without introducing unfamiliar additional information.

The misleading-illustration version used diagrams that were surface-similar to the conceptual illustrations but contained incorrect mechanistic information. For example, in the volcano text, the conceptual illustration depicted plate subduction and volcanic arc formation, whereas the misleading illustration retained the same general topic and diagrammatic format but incorrectly represented volcanic activity as originating from a large underground magma lake connected to the Earth’s core. In the glacier text, the misleading illustration similarly preserved the overall topic and visual style while misrepresenting key process relations involved in glacier movement. The plain-text version presented the same written content without any illustrations. Across the three versions, page layout, textual content, and reading order were kept as consistent as possible to minimize potential effects of formatting differences on learning outcomes. Representative screenshots of the volcano materials in the conceptual-illustration, misleading-illustration, and plain-text conditions are shown in Figure 1. All participant-facing materials were originally presented in Chinese, and the figure reproduces the original presentation format used in the experiment.

**Figure 1:**
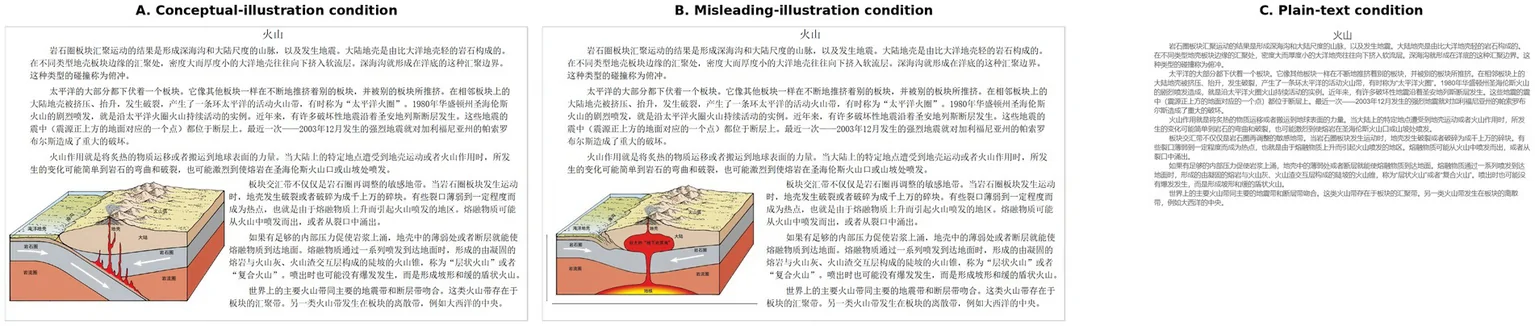
Representative screenshots of the volcano learning materials across the three instructional conditions. **(A)** Conceptual-illustration condition. **(B)** Misleading-illustration condition. **(C)** Plain-text condition. All participant-facing materials were originally presented in Chinese. Adapted with permission from Introduction to Geography, 11th edition (Simplified Chinese edition), Figure 3.8.

#### 2.2.2 Measures

The present study used a pre-test, language-related ability measures, learning outcome tests, and a cognitive load scale to assess participants’ prior knowledge, individual language abilities, learning performance, and subjective cognitive processing burden. Unless otherwise specified, binary-scored items were scored as 1 for a correct response and 0 for an incorrect response. For binary-scored measures, Cronbach’s α can be interpreted as equivalent to KR-20.

##### 2.2.2.1 Prior knowledge

The pre-test was used to assess participants’ knowledge of volcanoes and glaciers before learning. It consisted of 12 true-or-false items covering basic concepts, factual knowledge, and general scientific principles related to the two learning texts. Each correct response received 1 point, with a maximum score of 12. The items were developed based on the learning texts to ensure content alignment with the subsequent materials. In the present study, the pre-test showed acceptable internal consistency, Cronbach’s α = 0.756.

##### 2.2.2.2 Language-related abilities

Language-related abilities were assessed in terms of decoding ability and reading comprehension. Decoding ability was measured using a real-word/pseudoword judgment task based on the classic lexical decision paradigm. Lexical decision tasks requiring participants to discriminate real words from pseudowords or nonwords have been widely used to examine lexical recognition and word-level reading processes, including in Chinese readers (e.g., Tse et al., 2017). Participants were asked to judge whether each presented item was a real Chinese word or a pseudoword. This task was used to assess word-level lexical recognition and accuracy-based decoding-related processing. It included 60 items, with each correct judgment receiving 1 point and a maximum score of 60. The task showed good internal consistency in the present study, Cronbach’s α = 0.844. Participants obtained a mean score of 46.63 (SD = 5.46, range = 29–60), and only two participants (1.1%) achieved the maximum score.

Reading comprehension was measured using a deep cloze test. Cloze tests have long been used to assess individuals’ ability to integrate meaning and make semantic inferences based on discourse context. Deep cloze tests further emphasize the integration of contextual relations, discourse coherence, and implicit meaning, rather than relying solely on word recognition (Jensen and Elbro, 2022). The deep cloze test has also been adopted and further examined in subsequent studies of university students’ reading comprehension and situation-level text understanding (e.g., Bråten et al., 2025; Salmerón et al., 2022). Thus, this measure was suitable for assessing learners’ discourse-level reading comprehension. Participants were required to infer missing key words from the surrounding context. Each correct response received 1 point, and each incorrect response received 0 points. The deep cloze test showed good internal consistency in the present study, Cronbach’s α = 0.839.

##### 2.2.2.3 Learning outcomes

The learning outcome test included inference and retention items, which were used to assess learners’ deeper understanding and surface-level retention of the scientific texts, respectively. The item design followed prior text comprehension and science learning research that distinguishes inference questions from retention questions (Wiley et al., 2005). Each scientific topic included five inference items and five retention items, resulting in 20 post-test items across the two texts. Inference items required participants to integrate information distributed across the text and apply what they had learned to new problem contexts, thereby mainly reflecting situation model construction and learning transfer. Retention items required participants to recognize or recall facts, terms, and key information explicitly presented in the texts, thereby mainly reflecting surface-level information retention. Each correct response received 1 point, and each incorrect response received 0 points. In the present study, the internal consistency was acceptable for the inference items, Cronbach’s α = 0.716, the retention items, Cronbach’s α = 0.721, and the overall learning outcome test, Cronbach’s α = 0.738.

##### 2.2.2.4 Cognitive load

Cognitive load was measured using a multidimensional cognitive load scale. The items were adapted from the cognitive load measurement instrument developed by Leppink et al. (2013) and were modified to fit the scientific text learning context of the present study. Such scales typically assess learners’ subjective processing experience during learning across dimensions such as intrinsic cognitive load, extraneous cognitive load, and germane cognitive load (Klepsch et al., 2017; Leppink et al., 2013). The scale used in this study covered perceived material complexity, clarity of explanation, information conflict or confusion, and perceived learning support. Items were rated on a Likert-type scale, with higher scores indicating greater overall cognitive load reported by learners. In the present study, the cognitive load scale showed high internal consistency, Cronbach’s α = 0.910.

### 2.3 Procedure

The experiment was conducted online using Credamo (见数), a web-based research platform that supports the presentation of experimental materials and online data collection. Participants accessed the experiment through a web link and completed all tasks within the platform, with the entire experiment taking approximately 20 min. The platform was used to present the learning materials, administer the subsequent measures, and record participants’ responses. At the beginning of the experiment, participants read the instructions and completed a demographic questionnaire, including age, gender, and educational background. They then completed the pre-test, which assessed their prior knowledge of volcanoes and glaciers.

After the pre-test, participants completed the real-word/pseudoword judgment task and the deep cloze test, which measured decoding ability and reading comprehension, respectively. During the learning phase, participants were randomly assigned to one of three instructional conditions: conceptual illustration, misleading illustration, or plain text. The instructions asked participants to carefully read two natural science texts and informed them that they would complete related tests after reading.

Participants read the volcano and glacier texts sequentially on a computer screen. To control for order effects, the presentation order of the two texts was counterbalanced across participants: half of the participants read the volcano text first followed by the glacier text, whereas the other half read the texts in the reverse order. All texts were presented on standardized pages. Immediately after the learning phase, participants completed the learning outcome test. The test included inference and retention items. Inference items assessed participants’ ability to transfer, integrate, and apply the learned knowledge, whereas retention items assessed their retention and recognition of key information explicitly presented in the texts. Participants then completed the cognitive load scale to retrospectively report their subjective cognitive burden during the learning phase. The scale was administered after the learning outcome test to avoid prompting participants to consciously reflect on the difficulty of the learning materials before completing the outcome measures, which might have influenced their subsequent test performance. After all tasks were completed, the researchers screened the data for validity and provided compensation to participants who met the inclusion criteria.

### 2.4 Data analysis

Descriptive statistics and reliability analyses were first conducted for the main variables. Means, standard deviations, and Cronbach’s α were calculated for the pre-test, decoding ability, reading comprehension, learning outcome measures, and cognitive load scale to evaluate internal consistency. Correlation analyses were then performed to examine the basic associations among prior knowledge, language-related abilities, learning outcomes, and cognitive load.

To examine the main effect of instructional condition on learning outcomes, analyses of covariance were conducted. Instructional condition was treated as a between-subjects factor, and inference score, retention score, and overall learning score were entered as dependent variables in separate models. Pre-test score and other relevant covariates were controlled. Overall learning score was calculated as the sum of inference and retention scores. When significant group effects were observed, Holm-corrected *post hoc* comparisons were conducted to compare differences among instructional conditions. Effect sizes for main effects were reported using partial η^2^.

To examine whether language-related abilities moderated the effects of instructional condition on learning outcomes, moderation models were further constructed. Language-related abilities were divided into decoding ability and reading comprehension, both of which were included in the models. Each model included instructional condition, decoding ability, reading comprehension, and their interaction terms with instructional condition, while controlling for pre-test score. Continuous variables were standardized before entering the interaction models. For significant interactions, simple slope analyses were conducted, and predicted learning scores were estimated for each instructional condition at low (−1 SD), mean, and high (+1 SD) levels of the moderator.

To test whether cognitive load explained the negative effects of misleading illustrations on learning outcomes, mediation analyses were conducted. Instructional condition was entered as the independent variable, cognitive load as the mediator, and inference score, retention score, and overall learning score as dependent variables in separate models. The plain-text condition was used as the reference group, and pre-test score, decoding ability, and reading comprehension were controlled. Indirect effects were tested using a bootstrap procedure with 5,000 resamples, and 95% bootstrap confidence intervals were reported. An indirect effect was considered significant when the confidence interval did not include zero.

Finally, item-level mixed-effects models were conducted to examine whether the main findings were robust to the scoring method and individual item difficulty. Because the learning items were scored dichotomously as correct or incorrect, binomial logistic mixed-effects models were used. Item-level accuracy was entered as the dependent variable. Instructional condition, item type, reading comprehension, and their interaction terms were included as fixed effects, with pre-test score, decoding ability, and learning topic controlled. Random intercepts for participants and items were included. Items that all participants answered correctly and therefore showed no response variation were excluded from the item-level model only; all 20 items were retained in the pre-specified aggregate scores used in the primary analyses.

## 3 Results

### 3.1 Descriptive statistics and preliminary analyses

The final sample included 179 learners, who were assigned to one of three instructional conditions: conceptual illustration (*n* = 59), plain text (*n* = 60), or misleading illustration (*n* = 60). Descriptive analyses showed no significant differences among the three groups in pre-test scores or reading comprehension, indicating that the baseline levels were comparable after random assignment. Specifically, the group difference in pre-test scores was not significant, *F*(2, 176) = 1.12, *p* = 0.327. The group difference in reading comprehension was also not significant, *F*(2, 176) = 1.29, *p* = 0.279. In addition, age did not differ significantly across groups, *F*(2, 176) = 1.84, *p* = 0.162.

As shown in Tables 1, 2, the conceptual-illustration group showed higher inference, retention, and overall learning scores than the plain-text and misleading-illustration groups, whereas the misleading-illustration group showed the lowest performance. In contrast, cognitive load showed the opposite pattern: it was lowest in the conceptual-illustration group (59.15 ± 7.56), intermediate in the plain-text group (60.65 ± 8.86), and highest in the misleading-illustration group (65.17 ± 8.34). Correlation analyses further showed that reading comprehension was positively associated with inference, retention, and overall learning scores (*r* = 0.227, 0.307, and 0.347, respectively), suggesting that learners with higher reading comprehension generally achieved better learning outcomes. Multidimensional cognitive load was negatively associated with inference, retention, and overall learning scores (*r* = −0.291, −0.285, and −0.374, respectively), indicating that higher cognitive load was related to poorer learning outcomes. In addition, inference and retention scores were positively correlated (*r* = 0.184).

**Table 1:** Descriptive statistics by experimental condition.

Condition	*n*	Age	Pre-test score	Language comprehension	Inference score	Retention score	Overall learning score	Cognitive load
Conceptual illustration	59	20.95 (1.54)	8.56 (1.85)	26.24 (3.03)	7.46 (1.28)	7.44 (1.45)	14.90 (1.95)	59.15 (7.56)
Plain text	60	21.08 (1.53)	9.05 (1.88)	25.60 (3.05)	7.15 (1.42)	7.25 (1.56)	14.40 (2.19)	60.65 (8.86)
Misleading illustration	60	21.47 (1.52)	8.72 (1.74)	25.37 (3.10)	6.40 (1.79)	6.63 (1.69)	13.03 (2.74)	65.17 (8.34)

**Table 2:** Correlations among study variables.

Variable	1	2	3	4	5	6
1. Pre-test score	1	-	-	-	-	-
2. Language comprehension	0.212^**^	1	-	-	-	-
3. Inference score	0.169^*^	0.227^**^	1	-	-	-
4. Retention score	0.147^*^	0.307^***^	0.184^*^	1	-	-
5. Overall learning score	0.205^**^	0.347^***^	0.764^***^	0.774^***^	1	-
6. Cognitive load	−0.096	−0.196^**^	−0.291^***^	−0.285^***^	−0.374^***^	1

Values are Pearson’s r (two-tailed; *N* = 179). Significance markers are based on unadjusted *p* values: ^*^p < 0.05, ^**^p < 0.01, ^***^p < 0.001.

### 3.2 Main effects of instructional condition on learning outcomes

To examine the main effects of instructional condition on learning outcomes, ANCOVA models were conducted separately for inference score, retention score, and overall learning score, with pre-test score and reading comprehension entered as covariates. After controlling for these covariates, instructional condition significantly predicted inference score, *F*(2, 174) = 7.11, *p* = 0.001, η*_p_*^2^ = 0.076; retention score, *F*(2, 174) = 3.56, *p* = 0.030, η*_p_*^2^ = 0.039; and overall learning score, *F*(2, 174) = 9.70, *p* < 0.001, η*_p_*^2^ = 0.100. Adjusted means showed that the conceptual-illustration group scored higher than the plain-text and misleading-illustration groups across the three learning outcomes, whereas the misleading-illustration group showed the lowest overall performance.

Holm-corrected *post hoc* comparisons further clarified these differences (see Figure 2). For inference score, the misleading-illustration group scored significantly lower than the plain-text group, adjusted mean difference = −0.69, 95% CI [−1.22, −0.16], Holm-corrected *p* = 0.023, and also significantly lower than the conceptual-illustration group, adjusted mean difference = −1.00, 95% CI [−1.54, −0.46], Holm-corrected *p* = 0.001. The difference between the conceptual-illustration and plain-text groups was not significant, adjusted mean difference = 0.31, 95% CI [−0.23, 0.85], Holm-corrected *p* = 0.259.

**Figure 2:**
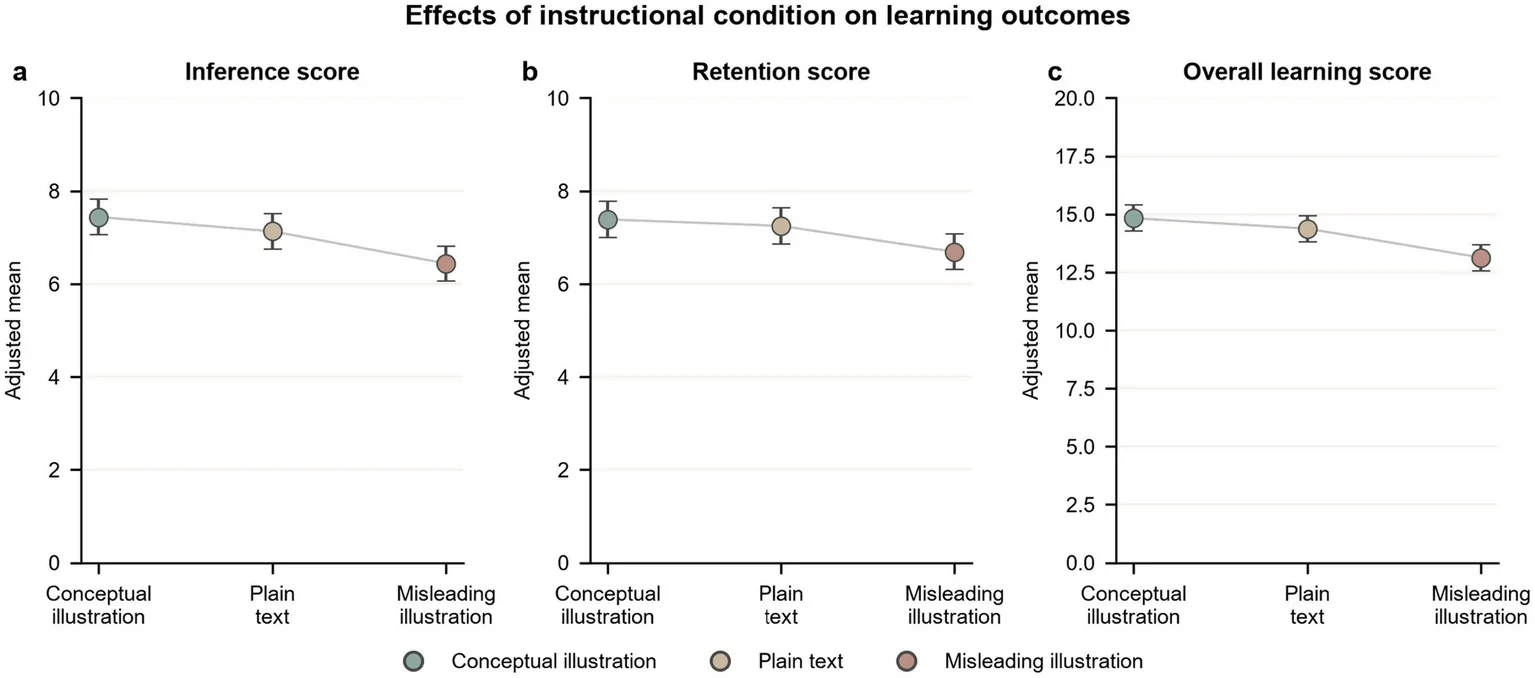
Main effects of instructional condition on learning outcomes. **(a)** Inference score. **(b)** Retention score. **(c)** Overall learning score. Points indicate adjusted means estimated from ANCOVA models controlling for pre-test score and language comprehension; error bars indicate 95% confidence intervals.

For retention score, the conceptual-illustration group scored significantly higher than the misleading-illustration group, adjusted mean difference = 0.70, 95% CI [0.15, 1.25], Holm-corrected *p* = 0.039. The misleading-illustration group scored lower than the plain-text group, but this difference did not remain significant after Holm correction, adjusted mean difference = −0.56, 95% CI [−1.10, −0.02], Holm-corrected *p* = 0.088.

For overall learning score, the misleading-illustration group scored significantly lower than the plain-text group, adjusted mean difference = −1.25, 95% CI [−2.03, −0.47], Holm-corrected *p* = 0.004, and also lower than the conceptual-illustration group, adjusted mean difference = −1.70, 95% CI [−2.49, −0.91], Holm-corrected *p* < 0.001. Overall, the disadvantage of the misleading-illustration condition was relatively stable for inference and overall learning performance, whereas its effect on retention was weaker.

### 3.3 Moderating effects of decoding ability and reading comprehension

The present study further distinguished language-related abilities into decoding ability and reading comprehension and examined whether they differentially moderated the effects of instructional condition on learning outcomes. The models included instructional condition, decoding ability, reading comprehension, and the interaction terms between instructional condition and each language-related ability, while controlling for pre-test score.

The results showed that the interaction between reading comprehension and instructional condition was relatively stable, as shown in Figure 3. Reading comprehension significantly moderated the effects of instructional condition on inference score, *F*(2, 169) = 4.05, *p* = 0.019, ηp^2^ = 0.046; retention score, *F*(2, 169) = 9.96, *p* < 0.001, ηp^2^ = 0.105; and overall learning score, *F*(2, 169) = 10.68, *p* < 0.001, ηp^2^ = 0.112. In contrast, the interactions between decoding ability and instructional condition were not significant for inference score, *F*(2, 169) = 1.86, *p* = 0.159, ηp^2^ = 0.022; retention score, *F*(2, 169) = 0.74, *p* = 0.479, ηp^2^ = 0.009; or overall learning score, *F*(2, 169) = 2.06, *p* = 0.131, ηp^2^ = 0.024.

**Figure 3:**
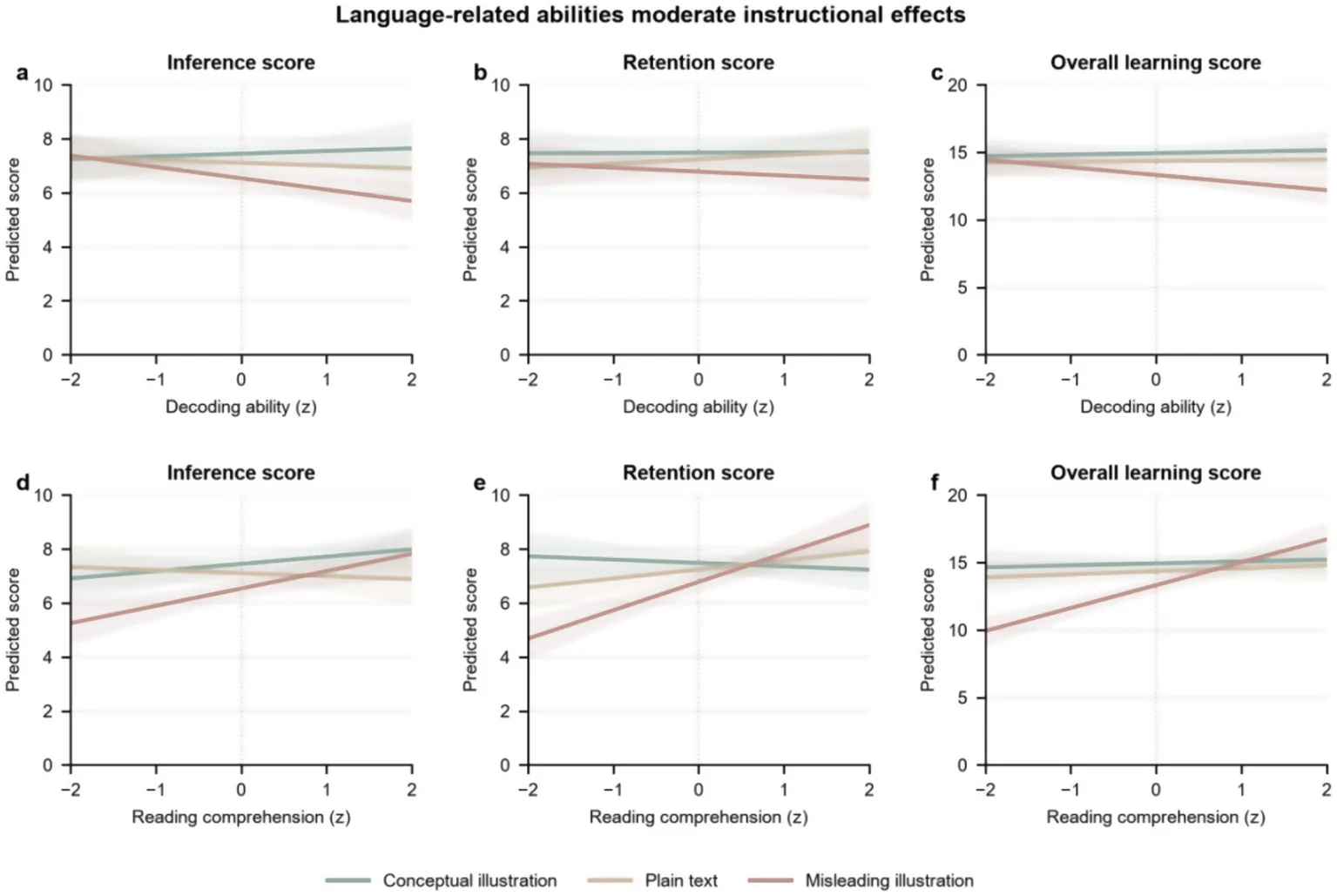
Moderation of instructional effects by language-related abilities. **(a–c)** Model-predicted inference, retention, and overall learning scores, respectively, across levels of decoding ability. **(d–f)** Model-predicted inference, retention, and overall learning scores, respectively, across levels of reading comprehension. Lines show model-predicted scores from linear models controlling for pre-test score; shaded bands indicate 95% confidence intervals. Decoding ability and reading comprehension were standardized before model estimation.

Simple slope analyses further indicated that reading comprehension primarily buffered the learning disadvantage associated with misleading illustrations. At low levels of reading comprehension (−1 SD), the predicted overall learning score in the misleading-illustration condition was 11.63, which was lower than that in the conceptual-illustration condition (14.79) and the plain-text condition (14.13). At high levels of reading comprehension (+1 SD), however, the predicted overall learning score in the misleading-illustration condition increased to 15.02, approaching the scores in the conceptual-illustration condition (15.08) and the plain-text condition (14.57). A similar pattern was observed for inference and retention scores. In the misleading-illustration condition, the predicted inference score increased from 5.89 at low reading comprehension to 7.18 at high reading comprehension, and the predicted retention score increased from 5.73 to 7.84. By contrast, the simple slope pattern for decoding ability did not show a comparable protective effect. In the misleading-illustration condition, the predicted overall learning score changed from 13.89 at low decoding ability to 12.75 at high decoding ability, showing no recovery as decoding ability increased.

These findings suggest that, in the present university sample, higher-level reading comprehension showed a more stable buffering role than accuracy-based decoding ability. This pattern is consistent with the idea that individual abilities may influence learners’ resistance to misleading visualizations, while further indicating that not all language-related abilities provide the same protection. Higher-level discourse comprehension, text–picture integration, and monitoring of inconsistent information may be more critical moderators.

### 3.4 Mediating role of cognitive load in the effects of misleading illustrations on learning outcomes

The mediation models treated instructional condition as the independent variable, cognitive load as the mediator, and inference score, retention score, and overall learning score as dependent variables in separate models. The plain-text condition was used as the reference group, and pre-test score, decoding ability, and reading comprehension were controlled.

The results showed that instructional condition significantly predicted cognitive load, *F*(2, 173) = 7.65, *p* < 0.001, ηp^2^ = 0.081. Adjusted means indicated that cognitive load was highest in the misleading-illustration condition and lowest in the conceptual-illustration condition. Further analyses showed that cognitive load negatively predicted learning outcomes. Specifically, cognitive load significantly predicted inference score, *b* = −0.034, *p* = 0.012; retention score, *b* = −0.035, *p* = 0.011; and overall learning score, *b* = −0.069, *p* < 0.001. These results indicate that higher cognitive load was associated with poorer learning performance.

As shown in Figure 4, bootstrap mediation analyses further indicated that, compared with the plain-text condition, misleading illustrations produced significant negative indirect effects on learning outcomes through increased cognitive load. Specifically, the indirect effect was significant for inference score, indirect effect = −0.147, 95% CI [−0.319, −0.024]; retention score, indirect effect = −0.152, 95% CI [−0.367, −0.015]; and overall learning score, indirect effect = −0.299, 95% CI [−0.611, −0.061]. In contrast, none of the indirect effects of conceptual illustrations relative to the plain-text condition reached significance.

**Figure 4:**
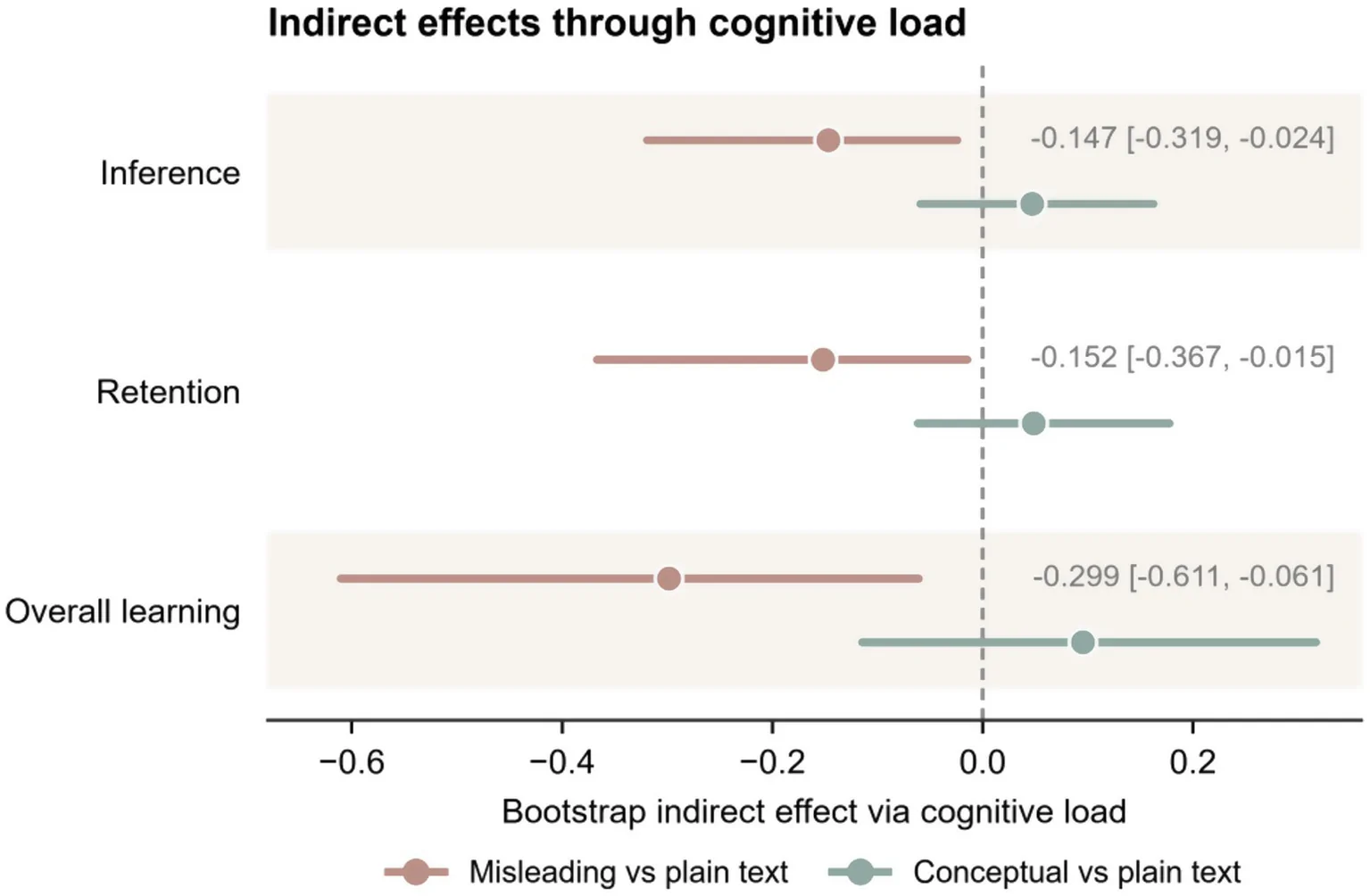
Mediating role of cognitive load in the effects of misleading illustrations on learning outcomes. Points indicate estimated indirect effects, horizontal lines indicate 95% bootstrap confidence intervals, and the dashed vertical line indicates zero effect.

Overall, these results suggest that cognitive load mediated the effects of misleading illustrations on learning outcomes. Misleading illustrations may increase learners’ information-processing burden, thereby impairing inference, retention, and overall learning performance. By contrast, conceptual illustrations did not show a stable cognitive-load-mediated effect.

### 3.5 Item-level mixed-effects analysis

To examine whether the above findings were stable at the item level, item-level mixed-effects models were further conducted. Because all learning items were scored dichotomously as correct or incorrect, binomial logistic mixed-effects models were used. Item accuracy was entered as the dependent variable. Instructional condition, item type, reading comprehension, and their interaction terms were included as fixed effects, while pre-test score, decoding ability, and learning topic were controlled. Random intercepts for participants and items were included. The full long-format dataset contained 3,580 observations. Six items (I03, I07, I10, I12, I14, and I18) were answered correctly by all participants and therefore showed no response variation. These comprised three inference and three retention items, with three items from each learning topic. Accordingly, they were excluded only from the item-level model, leaving 14 discriminating items and 2,506 observations for model estimation. As a sensitivity check, internal consistency was recalculated after their exclusion. Reliability remained acceptable and slightly increased for the overall test (α = 0.738 to 0.755), inference items (α = 0.716 to 0.752), and retention items (α = 0.721 to 0.757).

The results showed that, as illustrated in Figure 5, the misleading-illustration condition still produced significantly lower item-level performance than the plain-text condition, OR = 0.50, *p* < 0.001. The observed accuracy rates showed the same pattern. For inference items, the accuracy rate in the misleading-illustration condition was 64.0%, lower than that in the plain-text condition (71.5%) and the conceptual-illustration condition (74.6%). For retention items, the accuracy rate in the misleading-illustration condition was 66.3%, also lower than that in the plain-text condition (72.5%) and the conceptual-illustration condition (74.4%). In addition, the interaction between misleading illustration and item type was only marginally significant, OR = 0.73, *p* = 0.060, suggesting that the negative effects of misleading illustrations were not limited to a specific item type but were observed across both inference and retention items.

**Figure 5:**
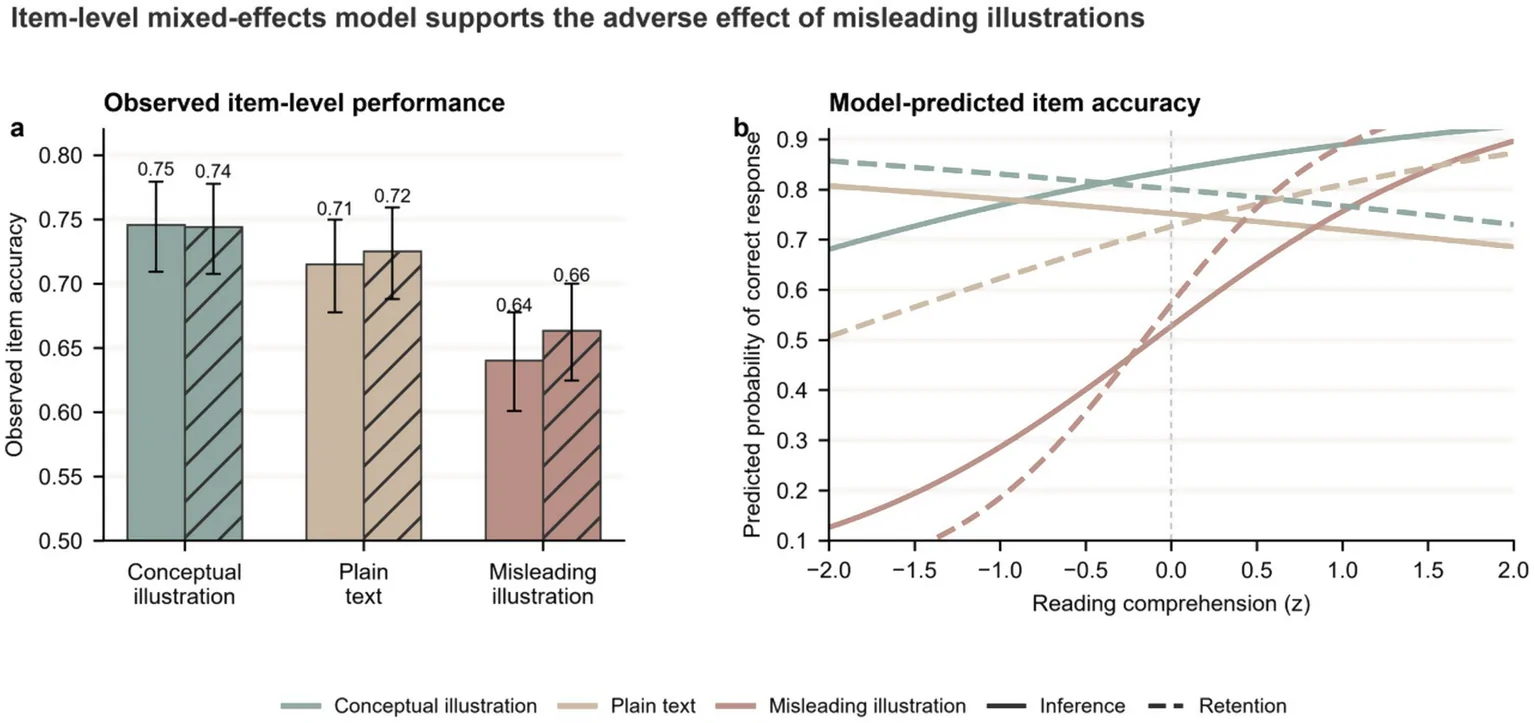
Item-level mixed-effects analysis of learning performance. **(a)** Observed item-level accuracy by instructional condition and item type. Bars indicate mean accuracy, and error bars indicate 95% Wilson confidence intervals. **(b)** Model-predicted probability of a correct response as a function of reading comprehension. Lines are fixed-effect predictions from the item-level logistic mixed-effects model controlling for pre-test performance, decoding ability, and topic, with random intercepts for participants and items.

Reading comprehension also significantly moderated the effect of misleading illustrations on item accuracy, OR = 3.65, *p* < 0.001. This finding was consistent with the total-score moderation analyses, indicating that higher reading comprehension buffered the learning disadvantage associated with misleading illustrations. The three-way interaction among misleading illustration, item type, and reading comprehension was not significant, OR = 0.90, *p* = 0.510, suggesting that the protective role of reading comprehension did not differ significantly between inference and retention items. Overall, the item-level mixed-effects models further supported the main conclusions of the present study: misleading illustrations had a stable negative effect on learning performance, and reading comprehension was an important individual-difference factor in resisting this effect.

## 4 Discussion

The present study showed that the role of instructional illustrations in scientific text learning does not depend simply on the presence of visual materials, but on whether the visual information supports learners in constructing accurate scientific explanations. Compared with the plain-text and conceptual-illustration conditions, misleading illustrations mainly impaired learners’ inference and overall learning performance, whereas retention showed a similar but weaker pattern. Further analyses indicated that the negative effects of misleading illustrations were associated with increased cognitive load and were moderated by reading comprehension. In contrast, decoding ability did not show a stable buffering effect. Item-level mixed-effects models further supported this pattern. Taken together, these findings suggest that illustrations that are surface-related but mechanistically inconsistent with the text may interfere with learners’ text–picture integration processes, turning visual materials from instructional support into a source of comprehension burden.

### 4.1 Negative effects of misleading illustrations on scientific text learning

Within the two short scientific expository texts examined in the present study, misleading illustrations were associated with lower learning performance, with the effects being most evident for inference and overall learning, whereas the effect on retention was comparatively weaker. In particular, misleading illustrations significantly reduced inference performance relative to both the plain-text and conceptual-illustration conditions, while the difference in retention between the misleading-illustration and plain-text conditions did not remain significant after correction for multiple comparisons. This pattern suggests that misleading illustrations may interfere more strongly with learners’ construction and application of an integrated understanding than with their memory for explicitly presented information. Prior research on multimedia learning and text–picture integration suggests that pictures are more likely to support learning when they help learners select, organize, and integrate key information (Mayer, 2021; Schnotz, 2014). Conversely, when pictures are inconsistent with the core content of the text or direct learners’ attention to weakly relevant information, they may interfere with situation model construction (Carney and Levin, 2002; Harp and Mayer, 1998; Mayer, 2005, 2021; Rey, 2012; Schnotz, 2014).

The stronger effect on inference than on retention can also be understood in terms of the different cognitive demands of these two types of learning outcomes. Retention items primarily required learners to recognize or recall information explicitly presented in the text, whereas inference items required them to integrate information distributed across the text and apply the underlying mechanism to new contexts. Therefore, misleading illustrations may be especially detrimental when successful performance depends on integrating and applying scientific relations, rather than simply retaining explicitly presented information.

This finding is also consistent with research on misleading visualizations. Previous studies have shown that visual presentation formats can influence learners’ judgments of informational relations, and that certain visual designs may systematically distort comprehension (Lauer and O’Brien, 2020; Lo et al., 2022; Pandey et al., 2015). Rho et al. (2026) further showed that misleading visual features reduce interpretation accuracy, that different types of misleading features vary in the strength of their effects, and that some misleading features continue to interfere with comprehension even among learners with relatively high data literacy. Although the materials used in the present study were not data charts but instructional illustrations embedded in scientific texts, both contexts involve the same core issue: how learners use visual cues to construct explanations of conceptual relations. The present study provides preliminary evidence that concerns about misleading visual information may also extend beyond data visualization to explanatory illustrations embedded in short scientific texts. Within the materials examined here, misleadingness depended not only on whether a picture was relevant to the topic, but also on whether it guided learners to construct conceptual or causal relations that deviated from the core mechanism described in the text.

It is also worth noting that the advantage of conceptual illustrations over plain text was not consistently significant. This suggests that the learning effects of illustrations are conditional. Haavisto et al. (2025) similarly noted that the effects of different material formats may depend on cognitive load, task demands, and learners’ reading abilities, indicating that visual or multimedia materials do not automatically lead to learning benefits. Taken together, the present findings suggest that the principal risk of mechanistically incorrect illustrations may lie in their interference with deeper inferential and integrative processing rather than with simple retention alone. Thus, illustrations in scientific texts should not be designed merely to increase visual richness. Instead, they should be aligned with the learning goals, textual structure, and scientific mechanisms. Only when illustrations accurately represent key relations and reduce comprehension demands are they likely to support scientific text learning.

### 4.2 Cognitive load as a mechanism underlying the effects of misleading illustrations on learning

Participants in the misleading-illustration condition reported the highest level of cognitive load, and cognitive load was negatively associated with learning performance. The mediation analyses further showed that, compared with the plain-text condition, misleading illustrations impaired learning performance partly by increasing cognitive load. This finding indicates that the effects of misleading illustrations were reflected not only in final learning outcomes, but also in learners’ subjective processing burden during learning.

This pattern can be explained by cognitive load theory. According to this theory, learners have limited working memory resources, and the complexity, presentation format, and irrelevant information in learning materials can influence the cognitive resources available for understanding and integrating core content (Paas et al., 2003; Sweller, 1988; Sweller et al., 2011). In multimedia learning, when visual materials clearly represent key relations in the text, they may reduce learners’ search, inference, and integration costs. However, when visual materials do not effectively support text comprehension and instead introduce additional demands for searching, judging, or integrating information, they may increase extraneous cognitive load and undermine learning performance (Klepsch et al., 2017; Leppink et al., 2013; Mayer, 2021; Mayer and Moreno, 2003). The misleading illustrations in the present study may have produced such additional processing demands. Learners had to understand the text while also judging the relation between the illustration and the text, and reconciling the verbal explanation with the visual cues. This additional processing may have consumed cognitive resources that could otherwise have been used to understand the core scientific content, thereby impairing inference and overall learning performance.

This interpretation is also consistent with previous multimedia-learning research showing that the design and composition of visual materials can influence learners’ cognitive load and learning outcomes. For example, studies on seductive details have shown that task-irrelevant or competing information can alter cognitive processing and learning, particularly when cognitive resources are already taxed (Park et al., 2015a, 2015b). Similarly, decorative visual elements have been found to increase extraneous cognitive load and impair subsequent recall (Pink and Newton, 2020). Haavisto et al. (2025) likewise showed that differences in material format can influence cognitive load and learning outcomes. Extending this work in a text-based learning context, the present study further suggests that the quality of visual materials can also shape learners’ processing burden. In other words, cognitive load may arise not only from reading itself, but also from inconsistencies between textual and visual information and from the interpretive costs imposed by misleading visual cues. For scientific text learning, therefore, illustration design should not only ensure the accuracy of visual information, but also minimize unnecessary interpretive demands.

### 4.3 The buffering role of reading comprehension from the perspective of the simple view of reading

Reading comprehension showed a relatively stable moderating role in the relationship between instructional condition and learning performance. It significantly moderated the effects of instructional condition on inference, retention, and overall learning scores. In contrast, the moderating effects of decoding ability were not significant. Thus, in the present university sample, reading comprehension emerged as a more stable moderator of the effects of misleading illustrations than accuracy-based decoding ability.

This finding can be understood from the perspective of the simple view of reading. According to this view, reading comprehension is jointly determined by decoding ability and linguistic comprehension, but these two components play different roles in reading processes (Gough and Tunmer, 1986; Hoover and Gough, 1990). Decoding ability primarily helps learners recognize written words and enter text-based processing, whereas reading comprehension involves the integration of sentence meaning, discourse coherence, and implicit relations. Haavisto et al. (2025) also showed that decoding ability and reading comprehension may affect learning outcomes and cognitive load in different ways. In the present context of scientific text learning, decoding ability provides an important foundation for understanding written materials. However, the present findings suggest that higher-level reading comprehension may be more closely related to learners’ ability to evaluate whether an illustration is consistent with the mechanism described in the text.

The difficulty posed by misleading illustrations was not simply whether learners could “read the words,” but whether they could identify the main ideas of the text, recognize core causal relations, and evaluate whether the visual information was consistent with the textual mechanism. This process relies more on discourse-level meaning construction, situation model formation, text–picture integration, and monitoring of inconsistent information than on word recognition alone (Kintsch, 1998; Mayer, 2021; Perfetti and Stafura, 2014; Schnotz, 2014). Learners with stronger reading comprehension may be better able to construct a stable situation model from the text and use it to screen, revise, or suppress misleading visual cues. In contrast, learners with weaker reading comprehension may be more likely to rely on surface-level pictorial information or weakly relevant visual cues, leading to interpretations that deviate from the text. This interpretation is also consistent with research on misleading visualizations, which suggests that higher-level interpretive and evaluative abilities can help reduce the impact of visual misinformation, although such protection still depends on the specific nature of the visual cues (Driessen et al., 2022; Rho et al., 2026).

The simple slope results further supported this interpretation. When reading comprehension was low, the misleading-illustration group showed substantially lower overall learning performance than the plain-text and conceptual-illustration groups. However, when reading comprehension was high, the predicted performance of the misleading-illustration group increased and approached that of the other two conditions. By contrast, decoding ability did not show a comparable protective pattern in the present sample. This pattern suggests that different language-related abilities may play different roles in text-picture integration (Cain and Oakhill, 2007; Oakhill and Cain, 2012). Higher-level reading comprehension may serve as an important resource for reducing the influence of misleading visual information, whereas no comparable buffering effect was detected for accuracy-based decoding ability in the present university sample.

This finding extends the explanatory scope of the simple view of reading in multimodal learning contexts. The simple view of reading has typically been used to explain how learners derive meaning from written text. The present study suggests that, in scientific texts containing misleading illustrations, reading comprehension may also support the monitoring of text–picture consistency. Stronger reading comprehension not only helps learners understand the text, but may also help them construct a stable situation model and use it to filter, revise, or inhibit erroneous visual cues. In contrast, although decoding ability is necessary for entering text-based processing, the present study did not detect a comparable protective effect of accuracy-based decoding ability against mechanistic visual misinformation.

### 4.4 Item-level analysis supports the robustness of the findings

The item-level mixed-effects models further supported the conclusions reported above. After controlling for participant-level and item-level differences, the probability of a correct response remained lower in the misleading-illustration condition than in the plain-text condition. The protective role of reading comprehension was also supported at the item level. These findings suggest that the negative effects of misleading illustrations were not driven solely by particular items or by the way total scores were aggregated. At the same time, the item-level results provide additional evidence for the robustness of the present findings regarding misleading illustrations and reading comprehension.

### 4.5 Instructional implications

The present findings provide preliminary implications for the design of explanatory illustrations in scientific texts involving causal or mechanistic content. First, instructional illustrations should not be designed solely on the basis of topical relevance or visual appeal. For scientific texts that involve complex causal mechanisms, the key function of illustrations should be to help learners identify relations among concepts and construct coherent mechanistic representations. If an illustration is only surface-related to the topic, or if it implies information that is inconsistent with the textual explanation, it may increase learners’ processing burden and impair learning.

Second, illustration design should take learners’ differences in reading comprehension into account. Learners with weaker reading comprehension were more vulnerable to misleading illustrations, suggesting that they may rely more heavily on external visual cues and may have greater difficulty maintaining the main textual explanation when textual and visual information are inconsistent. Therefore, for learners with weaker reading comprehension, instructional materials should minimize weakly relevant or potentially misleading visual information. Clear labels, step-by-step presentations, and explicit text–picture correspondence cues may help reduce integration difficulty.

Finally, the findings also have implications for adaptive learning systems. Digital learning environments can provide differentiated text–picture scaffolds based on learners’ reading comprehension. For learners with weaker reading comprehension, systems may provide more explicit correspondence cues between text and illustration, labels for key mechanisms, or alerts about potentially misleading visual cues. For learners with stronger reading comprehension, systems may provide more open comparison and reflection tasks to encourage active monitoring of text–picture consistency. In this way, illustrations can move beyond static visual elements and become instructional tools that support scientific understanding and reduce the risk of visual misinformation.

### 4.6 Limitations and future directions

Several limitations should be noted. First, the learning materials were limited to two relatively short scientific expository texts on volcanoes and glaciers. Both texts were designed with comparable expository structures and corresponding process illustrations, which supported experimental control across conditions. However, these materials represent only a narrow subset of scientific texts in terms of topic, length, complexity, and representational form. They provide evidence for the effects of misleading illustrations within the specific type of short scientific expository materials examined here. Future research should replicate these effects using longer and more complex texts, a wider range of scientific domains, and different types of explanatory illustrations before broader conclusions are drawn.

Second, cognitive load was assessed solely through a self-report questionnaire. Although the scale showed high internal consistency and captured learners’ subjective processing experience, self-reported cognitive load may be susceptible to response bias and may also reflect factors unrelated to the experimental manipulation, such as fatigue, distraction, individual response tendencies, or other situational influences. Therefore, the higher cognitive load reported in the misleading-illustration condition cannot be attributed exclusively to the misleading illustrations, and the mediating role of cognitive load should be interpreted as reflecting participants’ perceived processing burden rather than a direct measure of cognitive resource consumption. In addition, questionnaire measures cannot capture moment-to-moment changes in cognitive processing during reading and text–picture integration. Future studies should combine self-report measures with more objective or process-sensitive indicators, such as eye tracking, response times, pupillometry, or reading-trajectory data, to more precisely determine whether misleading illustrations increase processing demands at points of text–picture inconsistency and how these processes contribute to subsequent learning outcomes.

Third, participants in the present study were university students with generally high levels of basic decoding ability. Although the lexical decision task showed good internal consistency and only 1.1% of participants achieved the maximum score, overall decoding accuracy was relatively high. Moreover, the task relied on response accuracy and may therefore have been less sensitive to subtle individual differences in decoding efficiency among skilled adult readers. Accordingly, the non-significant moderating effect of decoding ability should be interpreted cautiously and should not be taken as evidence that decoding is irrelevant to learning from illustrated texts. Future studies could employ more sensitive measures, such as timed lexical decision, word-reading fluency, or reaction-time-based indices.

Finally, the item-level analysis showed that some learning items had very high accuracy rates and lacked response variation. This suggests that future research should further optimize item difficulty and include more inference and transfer items that can distinguish different levels of understanding. Future work could also differentiate among types of misleading illustrations in greater detail, such as weakly relevant illustrations, illustrations with incorrect causal relations, and visually salient but conceptually irrelevant illustrations, to determine whether different forms of misleading visual information have distinct cognitive consequences.

## 5 Conclusion

This study examined the effects of misleading illustrations on learning from two short scientific expository texts and further explored the roles of cognitive load and language-related abilities. Within these materials, misleading illustrations were associated with poorer inference and overall learning performance than plain text and conceptual illustrations. Compared with plain text and conceptual illustrations, misleading illustrations mainly impaired learners’ inference and overall learning performance. This suggests that when illustrations are inconsistent with the text in key conceptual relations, process sequences, or causal mechanisms, visual information may shift from a scaffold for comprehension to a source of interference.

Cognitive load was found to be an important mechanism underlying this effect. Misleading illustrations may increase learners’ processing burden during text–picture searching, judgment, and integration, thereby consuming cognitive resources that could otherwise be used to construct scientific mechanisms. In addition, from the perspective of the simple view of reading, reading comprehension consistently buffered the negative effects of misleading illustrations, whereas decoding ability did not show a comparable protective role. This indicates that resisting erroneous visual cues depends not only on basic word recognition, but also on learners’ ability to integrate and monitor textual meaning, discourse relations, and causal mechanisms.

Overall, this study extends research on misleading visual information from data visualization to scientific text illustrations. Within the short scientific expository texts examined here, the findings provide initial evidence that mechanistically misleading illustrations can interfere with learning and highlight the potential importance of consistency between textual and pictorial representations. Broader generalization of these findings, however, requires further examination across different scientific domains, text lengths, and types of instructional illustrations.

## Data Availability

The raw data supporting the conclusions of this article will be made available by the authors, without undue reservation.
